# Prevalence and risk factors of epizootic lymphangitis in cart pulling horses and mules in Central and South Gondar zones, Amhara region, Ethiopia

**DOI:** 10.1016/j.heliyon.2022.e09939

**Published:** 2022-07-19

**Authors:** Amsalu Misgie Molla, Wudu Temesgen Jemberu, Tewodros Fentahun

**Affiliations:** a College of Agriculture and Environmental Sciences, Debre Tabor University, P. O. Box 272, Debre Tabor, Ethiopia; b International Livestock Research Institute, P.O. Box 5689, Addis Ababa, Ethiopia; c College of Veterinary Medicine and Animals Sciences, University of Gondar, P. O. Box 196, Gondar, Ethiopia

**Keywords:** Cart pulling equids, Epizootic lymphangitis, Ethiopia, *Histoplasma capsulatum*, Prevalence, Risk factors

## Abstract

Epizootic lymphangitis (EZL) is a chronic, contagious disease of equids caused by the fungus *Histoplasma capsulatum* var. *farciminosum*. The disease is often prevalent in cart pulling equids in developing countries. This study was undertaken in Central and South Gondar zones of Amhara region, Ethiopia with the objectives of estimating the prevalence of EZL in cart pulling horses and mules and identifying the risk factors of the disease. Two towns, one high altitude and one mid altitude, from each of the two zones were included for the study. A total of 528 cart pulling horses and mules from the selected towns were examined clinically, screened by physical examination based on clinical signs of the disease and confirmed by microscopic mycological examination of the causative agent. Potential risk factors data were collected through observation of cart animals and interviewing of cart owners and were statistically evaluated using mixed effect logistic regression. An overall EZL prevalence of 12.5% (95% CI: 9.9–15.6%) was found. The prevalence was 19.9% (95% CI: 15.8–24.8%) in horses and 5.8% (95% CI: 2.4–13.2%) in mules. The disease was prevalent in mid altitude towns but was not detected in high altitude cold towns of the study zones. The risk factor analysis revealed that sharing of harness, mingling of cart animals in cart stations, communal housing and pre-existing trauma wound were risk factors of EZL in cart pulling horses and mules. The study generally indicated that EZL is a prevalent problem in mid altitude towns that endangers the livelihood of the cart owners and wellbeing of cart pulling equids. This warrants the initiation of a control strategy mainly focusing on improving management of cart pulling equids related to the identified risk factors to ameliorate the EZL problem in the study area.

## 1 Introduction

Ethiopia has the largest equine population in Africa [1]. The country's equine population is estimated at 13.3 million composed of 2.15 million horses, 0.38 million mules and 10.79 million donkeys [2]. Working equids have vital roles in the Ethiopia's agricultural and transport systems; such as transport of farm products, fodder, firewood, water, agricultural inputs, construction, waste materials and people [3, 4, 5]. They are mainly used as draught and pack animals and also used for ploughing in some parts of the country [4]. Additionally, horse and mule drawn cart business has long been used as a source of income for significant proportion of urban population of the country [6, 7, 8].

Equines in Ethiopia are burdened with prevalent infectious and noninfectious health problems [9, 10]. The health problems and diseases affecting working equids and their productivity in Ethiopia have been assessed previously and health problems such as epizootic lymphangitis (EZL), colic, lameness, lip wound, parasites, African horse sickness, foot abscess, bloating, swollen legs, mouth lesion, harness related wounds and sores, anthrax, sarcoid, rabies, ocular disease, joint swelling have been reported [8, 10]. Among these, EZL is the most common problem in cart animals in Ethiopia [8, 11].

Epizootic lymphangitis is a contagious, chronic disease of equids, caused by a dimorphic fungus *Histoplasma capsulatum* var. *farciminosum*. The disease is transmitted through contact, by biting insects or inhalation of spores [12, 13]. Epizootic lymphangitis can be described clinically in four forms: cutaneous, ocular, respiratory, and mixed forms; and the cutaneous form is the most common [14, 15, 16]. The disease can be diagnosed based on the clinical examination of the lesions, microscopic examination of the yeast form of the fungus in pus, serological tests or skin hypersensitivity testing [13, 17, 18].

The disease is endemic in west, north, and north-east Africa, the Middle East, India, and the Far East [14, 19]. Most outbreaks occur in humid and hot climates; when large numbers of equids are stabled together for transportation needs, in military situations or racing [13]. The disease is currently prevalent in Ethiopia; with prevalence in horses ranging from 0 - 39% in various regions of the country [11, 20, 21].

There were few studies of EZL in Amhara National Regional State of Ethiopia. The available studies showed the problem is becoming a serious threat to the productivity and welfare of working equids, especially on cart pulling horses and mules in various towns of the region. Previous studies in the western part of the region by Mesafint *et al.* [21] in Gondar town and Meselu *et al.* [22] in Bahir Dar town revealed a total prevalence of 23.2% in cart horses and 32.84% in cart mules respectively. Prevalence of 32.5%, 21.7%, and 16.3% in Bati, Kemissie and Kombolcha respectively were reported by Ameni [11] in the eastern part of the Region.

The present study was conducted in the South and Central Gondar zones of the western part of the region where there is no data on the prevalence of the disease. The objectives of this study were to determine the prevalence of the EZL in the study areas and assess the potential risk factors that expose cart pulling horses and mules to the disease.

## 2 Materials and methods

### 2.1 Description of the study area

**Figure 1:**
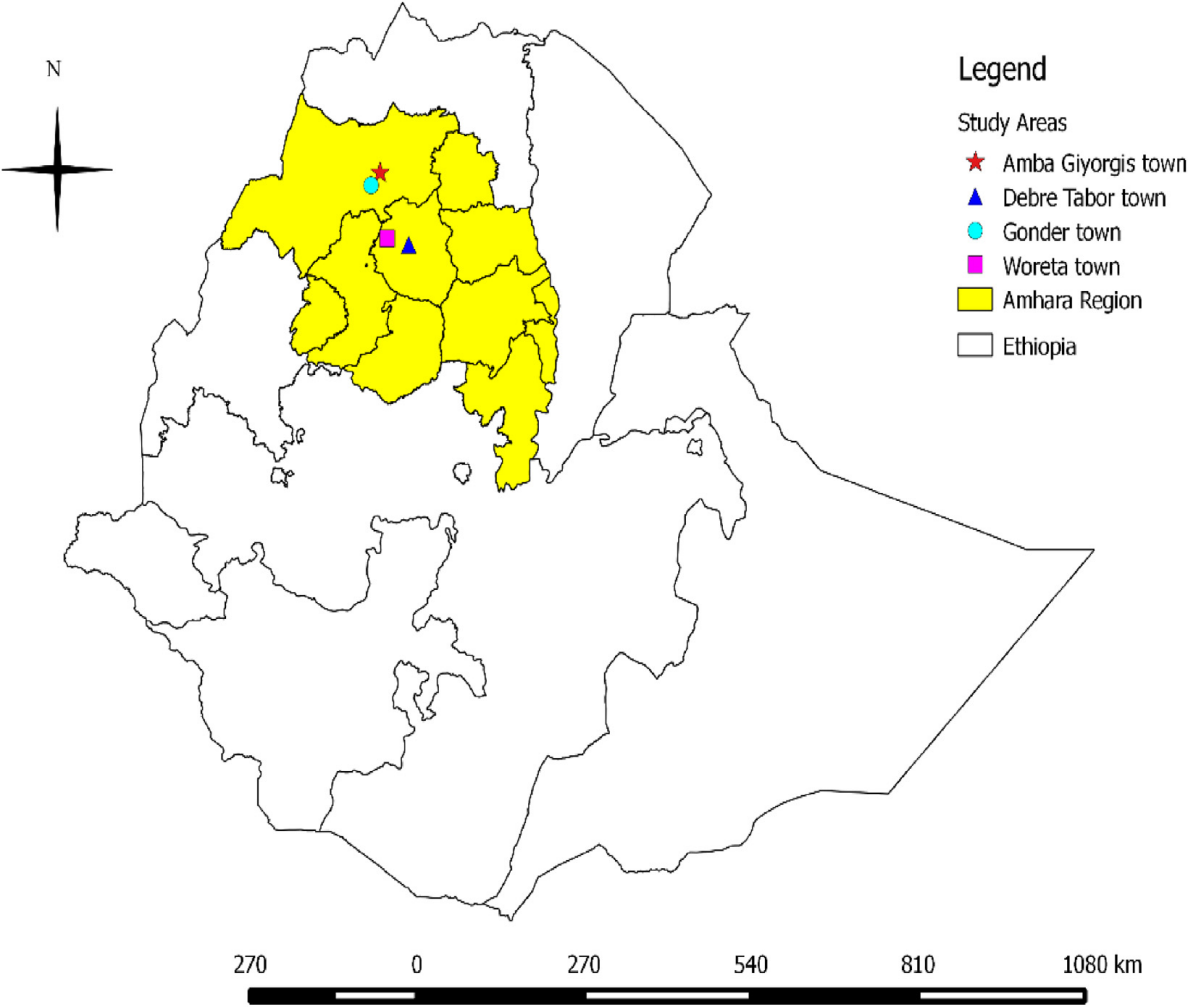
Map showing the study towns.

The study was conducted in four selected towns (Gondar and Amba Giorgis from Central Gondar zone, and Debre Tabor and Woreta from South Gondar zone) of Amhara regional state (Figure 1) in 2019.

Gondar town is found at 12º45′ North latitude and 37° 45′ East longitudes and an altitude of 2133 m above sea level (m.a.s.l.). It has an average annual temperature of 19.3°C, and mean annual rainfall of 1200 mm. The town administration has livestock population of 82,029 cattle, 2,695 sheep, 22,590 goats, 2,065 horses, 244 mules, 9,001 donkeys, 718, 256 poultry and 7, 254 beehive population [23]. The cart horse population in the town is about 1100 [24].

Amba Giorgis town is located at 12°46′ North latitude and 37°37′ East longitude. It has an elevation of 2779 m.a.s.l. with *dega* (cold) agro-climatic zone, average annual temperature of 14 °C; mean annual rain fall of 400–700 mm. The town has about 275 horses and 10 mules [25].

Debre Tabor town is located at 11°51′ North latitude and 38°1′ East longitude. It has an elevation of 2706 m.a.s.l. with *dega* and *Woina-dega* (temperate) agro-climatic zone, average annual temperature of 14.8 °C and mean annual rain fall of 1553.7 mm. The town has livestock population of 1769 cattle, 1881 sheep, 109 goats, 52 horses, 184 mules, 258 donkeys, 14828 poultry, 65 beehive and 89 swine populations [26]. The town has about 152 cart mules and 50 cart horses [27].

Woreta town on the other hand is found at 11°55’ North latitude and 37° 42' East longitude and at an altitude of 1828 m.a.s.l. with kola (hot) and Woina-dega agro-climatic zone. It has an average annual temperature of 20.3°C and mean annual rain fall of 1216.3 mm [28]. The town has livestock population of 2419 cattle, 485 sheep, 119 goats, 411 mules, 28 donkeys, 4250 Poultry and 17604 beehive populations [29]; and the town has about a cart mule population of 313 [28].

### 2.2 Study population and animals

The study was conducted on cart horses and mules population of the study towns. Cart horses and mules are those used for cart pulling in transportation of humans and goods. In Gondar town only horses, in Woreta town only mules, and in Amba Giorgis and Debre Tabor towns both horses and mules were used for cart pulling.

### 2.3 Study design and sampling strategy

The study was a cross-sectional study in which clinical and laboratory examinations of study animals were carried out to determine prevalence of EZL. Potential risk factors data were collected through observation and asking of animal owner during clinical examination.

A multistage cluster sampling was implemented to select the study cart horses and mules. Towns in the study zones were the primary sampling units, individual cart owners were secondary sampling units and individual cart pulling equids were tertiary sampling units. At stage one, four towns in the two zones (two towns in Central Gondar and two towns in South Gondar) were selected purposively to represent the two zones in terms of climate and also convenience of accessibility. Amba Giorgis town from Central Gondar zone and Debre Tabor town from South Gondar zone are high altitude and have cold climate whereas Gondar town from Central Gondar zone and Woreta town from South Gondar zone are mid altitude and have warmer climate. In stage two, individual cart owners from each town were selected using simple random sampling. The lists of cart animal owners of those selected towns were obtained from municipality or cart associations' offices. In stage three, all cart pulling horses and mules of the selected cart owners were included for the study. The selected cart owners were identified at cart stations, market area, veterinary clinics, and when not accessed in these sites, a home visit was made. The animals were subjected for clinical examination and clinically positive animals were sampled for laboratory examination and the owners were interviewed for different management practices that could potentially be risk factors for the disease.

### 2.4 Sample size determination

The sample size for determining the prevalence of the diseases and associated risk factors was calculated using the method described by Thrusfield [30] and as given by Eq. (1):

(1) n = (1.96)^2^ [Pexp (1-Pexp)] / e^2^

Where n is the required sample size, Pexp is the prevalence expected in the area and e is the margine of error.

A previous 23.2% prevalence determined in Gondar town by Mesafint *et al.* [21] was taken into consideration for calculating the sample size; therefore by using an expected disease prevalence of 23.2%, margine of error 5%, and confidence level of 95%, a sample size of 274 was determined, But the size of the total cart horse and mule population (N) in the study towns was only 1900 which was small relative to the sample size (n) determined (n ∗ 10 > N). So the calculated sample size was adjusted for finite study population using Eq. (2) [31] resulting adjusted sample size of 240.

(2) n = n_0_ / [1+ (n_0_ / N)]

Where n is the adjusted sample size, n_0_ is the initial sample size, N is study population.

The above sample size calculation is applicable for simple random sampling but the sampling strategy used in this study was multistage cluster sampling. So the calculated sample size was adjusted for multistage cluster sampling by using a rule of thumb of doubling the sample size calculated for simple random sampling [32]. Accordingly, the sample size was doubled to 480 (240∗2) and finally a 10% (0.1∗480 = 48) potential noncompliance rate was added for replacement and a sample size of 528 cart animals (396 cart horses and 132 cart mules) were used as final sample size. The total sample size was distributed among four study towns proportional to towns’ total horse and mule population. The average number of cart animal per cart owners was used to determine the number of cart owner to be included in each town for achieving the number of cart animals proportionally allocated for the towns. Accordingly, 79 cart animals out of the total of 285 carts animals in Amaba Georgis, 56 out of 202 in Deber Tabor, 306 out of 1100 in Gondar and 87 out of 313 in Woreta towns were sampled.

### 2.5 Data collection methods

#### 2.5.1 Clinical examination

For prevalence study, cart horses and mules were screened for EZL by physical examination based on clinical signs of the disease. Clinical case definition was set to categorize animals as either clinically positive or negative for EZL. If a horse or mule observed with one or more of the clinical signs such as appearance of freely movable cutaneous nodules and skin eruption mostly seen in extremities, chest wall and the neck and follow a lymphatic line, and/or with nodules which suppurate, discharge a thick yellow pus, ulcerating and spreading to neighboring lymph glands which becomes swollen and hard, was considered as clinically positive for EL and otherwise were recorded as negative.

During physical examination, all parts of the body of the animals including the nostril and eyes were visually examined and palpated for the presence of lesions of EZL especially for the presence of nodules or ulcers. Emphasis was given to the lymphatic vessels, lymph nodes and skin. Besides, the owners were enquired to recall any lesion, or nodules observed in their horses. When an animal is categorized clinically positive up on clinical examination, then the severity of the disease was graded as mild, moderate and severe based on criteria described in Annex 1 [33].

#### 2.5.2 Sample collection and laboratory examination

In horses and mules that were clinically categorized as positive, pus/exudate sample was collected from the lesions for further confirmation by microscopic mycological examinations.

Laboratory confirmation of the clinical cases was done using Gram staining of the collected sample [17]. Nodules (preferably intact nodules) selected and washed with soap and water, shaved, and disinfected with alcohol swab (70% ethanol) to remove surface contaminants for fine needle aspiration (FNA). FNA was performed using a standard syringe (5ml) and needle (22 gauges) [13, 17, 34].

Then aspirated pus sample was obtained aseptically from un-ruptured nodules (all cases had at least one unruptured nodules that were used for sampling) and used for microscopic mycological examination. Briefly the aspirated pus samples were smeared on clean glass slides, allowed to fix with methanol (2–3 min), and then stained directly with gram's stain and examined for the typical yeast form of the organism, which appeared as gram-positive, pleomorphic, ovoid to globose structures, approximately 2–5μm in diameter. Examination was done by using oil immersion at 100x magnification. Confirmation of the disease was based on this microscopic demonstration of *H. capsulatum* var. *farciminosum* in the gram stained smear (Annex 2).

#### 2.5.3 Risk factor data collection

During clinical examination and sampling of cart animals; the potential animal, management, environmental and owner related potential risk factor for the disease were recorded in prepared format (Annex 3) by observation and asking the cart owners. Potential risk factors included were species, age, presence of preexisting wounds, body condition, housing, feeding, grooming practice, harnessing, mingling with other cart animals, altitude of the town and education status of owners etc. The age of the horses and mules was asked from the owners and was categorized as young (≤6 years) and adult (>6 years) following on previous works [15, 21]. The body condition of the horses and mules was scored in scale of 1–9 as described in Annex 4 [35]. These body condition scores were categorized into three qualitative categories: scores 1–3 as poor, 4–6 as medium and 7–9 as good to facilitate the analysis. Grooming practice (washing and cleaning the cart animal) was categorized as ‘yes’ when there was the practice irrespective of the frequency and ‘no’ when there was no any grooming practice at all. Housing and feeding practice were categorized as ‘separate’ when cart animals were individually housed and feed and ‘shared’ when they were housed and feed together.

The study protocol was reviewed and approved by the Institutional Review Board of University of Gondar. Informed oral consent was obtained from cart owners to participate in the study.

### 2.6 Statistical analysis

The statistical analyses were carried out using STATA version 14 (Stata Corp. College Station, TX). The prevalence of EZL was calculated as a proportion of positives to the total number of animals examined.

Mixed effect multivariable logistic regression was employed to identify potential risk factors associated with EZL. Gram-stain status (positive or negative) was the dependent variable, the putative EZL risk factors were fixed effect predictor variables and town was a random effect predictor variable in the regeression analysis. Mixed effect logistic regression was used to account for the clustering expected within towns and cart owners that could arise from multistage cluster sampling method used in the data collection. But the clustering effect at cart owner level was ignored because of small cluster size (1.05 cart animals/cart owner) and hence only town was included as random effect variable.

The potential risk factors that were considered as predictor variables to the model were altitude, educational status of the owners, species, age and body condition; and management related variables such as housing/shelter, preexisting trauma wounds, harnessing practices, grooming practices and assembling with other cart animals. Collinearity among the predictor variables was checked by correlation matrix and then the two variables namely altitude and average annual temperature (^0^C) were found collinear with a predictor variable species, so these two variables were dropped from the model.

The remaining putative risk factors were analyzed by using the mixed effect multivariable logistic regression analysis. First the full model with all the predictor variables was run. The final model was fitted by backward elimination; first the least predictor (i.e. predictor having the largest P-value) was removed and then the model allowed running again. When removal of a predictor changed the coefficients of the remaining predictors by more than 30%, it was considered as a confounder and retained in the model [31]. The model was run again in the same manner until only statistically significant (P < 0.05) predictors and confounders were left, resulting in the final model.

Prevalence odds ratio (POR) was used to measure strength of associations of the predictor variables with the outcome variable. In all the analyses, the confidence level was held at 95% and P-value less than 0.05 was set for statistical significance. The study areas were mapped using GIS software, QGIS version 2.18.

## 3 Results

### 3.1 Prevalence and disease characterization

Out of the total of 528 cart animals (396 cart horses and 132 cart mules) clinically examined, 70 animals were diagnosed as positive for EZL. From the 70 clinically positive cases 66 (61 horses and 5 mules) of them were confirmed by laboratory demonstration of the causative yeast *H. capsulatum* var*. farciminosum* in gram stained aspiration pus smears (Annex 5) resulting in an overall prevalence of 12.50% (95% CI: 9.93–15.61%). The prevalence was variable among the study towns; the highest prevalence (19.93%) was recorded in Gondar town, and the lowest was in Amba Giorgis and Debre Tabor towns where no EZL positive animal was detected (Table 1). In the positive towns, prevalence in horses was 19.93% and the prevalence in mules was 5.75%.

**Table 1:** Prevalence of epizootic lymphangitis by the study towns.

Town district	No of animals Examined	Total Positive	Prevalence (%) 95% CI
Amba Giorgis Town	79	0	-
Debre Tabor Town	56	0	-
Gondar Town	306	61	19.93 (15.82, 24.81)
Woreta Town	87	5	5.75 (2.39, 13.16)
**Total**	**528**	**66**	**12.50 (9.93, 15.61)**

### 3.2 Distribution and characteristics of lesions

**Figure 2:**
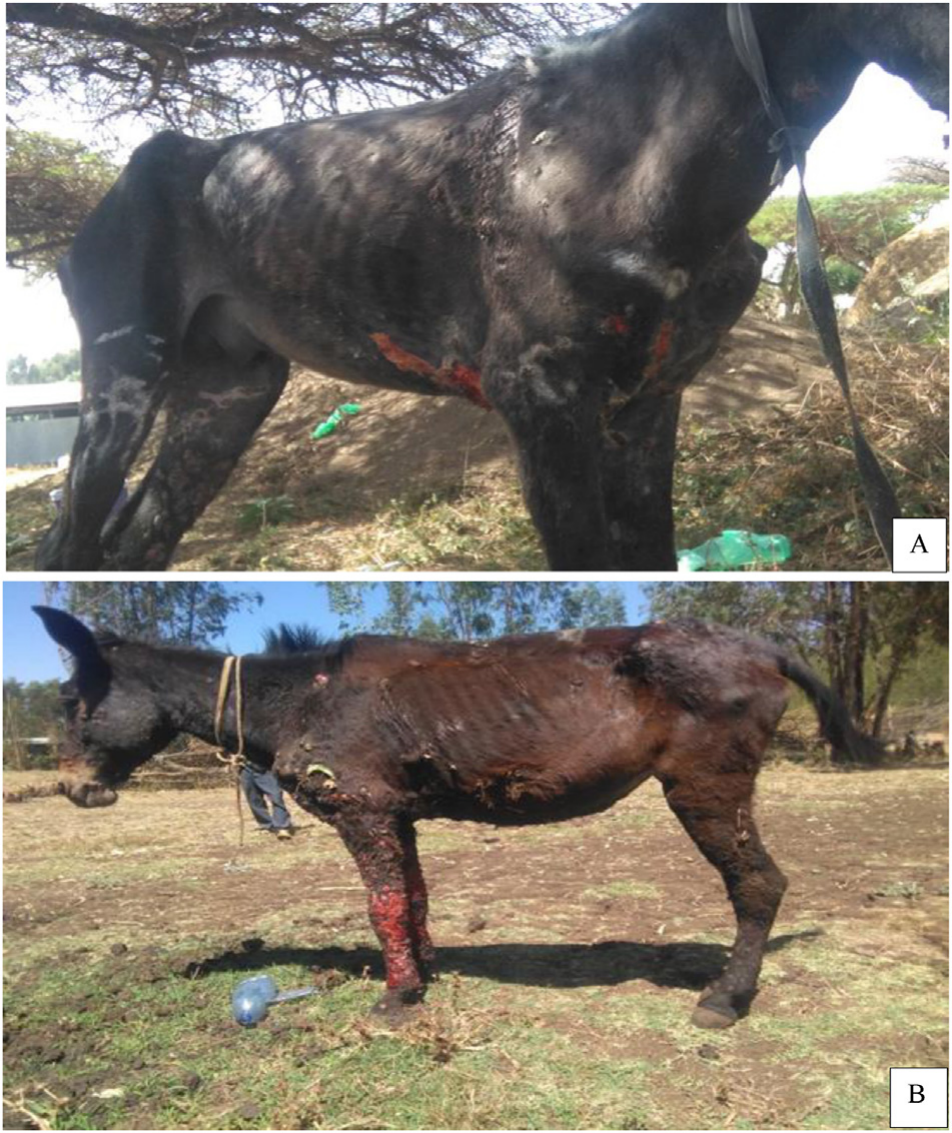
Cutaneous form of epizootic lymphangitis in cart horse **(A)** and Cart mule **(B)**.

**Figure 3:**
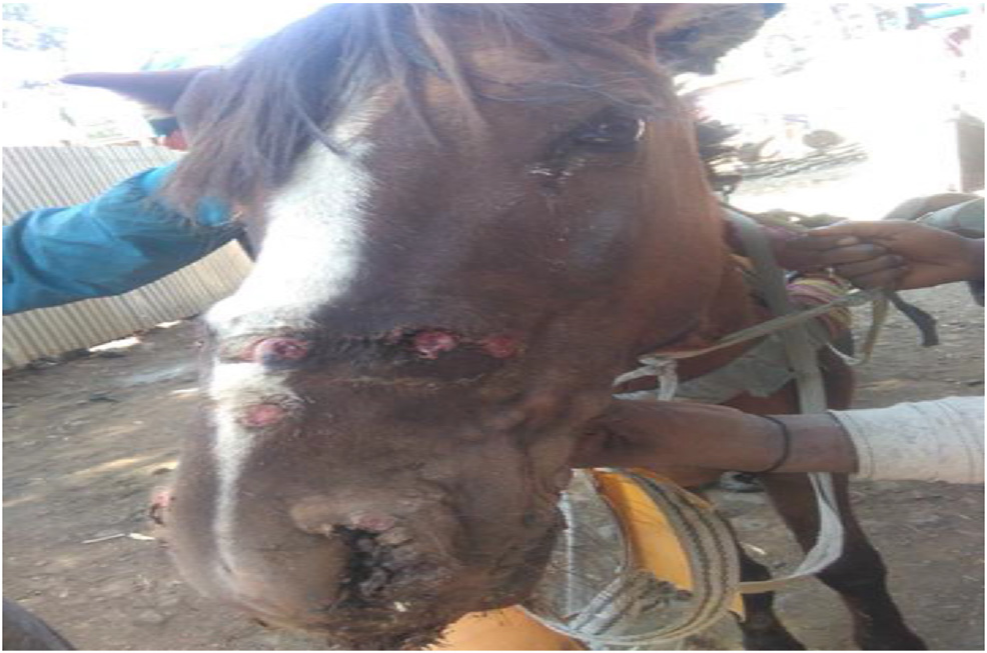
Cutaneous and respiratory form of epizootic lymphangitis in cart horse.

Among the 66 EZL positive cart animals, 65 cases (98.5%) were found with cutaneous form of the disease (Figure 2). One case was presented with both cutaneous and respiratory form of EZL in which multiple small nodules and ulcers in the external nares and muzzle, and mucopurulent nasal discharge was observed (Figure 3).

Among the diseased cart animals, the highest percentage (78.8%) of the EZL lesions were found on the limbs and the majority (39.4%) of the cases were presented with severe form of the disease (Table 2).

**Table 2:** Distribution on different body parts and severity of EZL lesion in cart pulling animals.

Lesion description	Frequency	Percentage
**Lesion Location**		
Limbs	52	78.79
Belly and/or sternum area	22	33.33
Chest wall	29	43.94
Neck region	18	27.27
Face	21	31.82
Perineum	12	18.18
Inguinal area	9	13.64
Back	4	6.06
**Lesion Severity**		
Mild	22	33.33
Moderate	18	27.27
Severe	26	39.39
Total	66	100.00

### 3.3 Risk factors

Potential risk factors that were evaluated for their association with prevalence of EZL using a mixed effect multivariable logistic regression model are presented in Table 3.

**Table 3:** The full model for the risk factor analysis of EZL in the four towns (n = 528).

Risk factor	Total Sampled	Number of Positive	Prevalence (%)	POR (95% CI)	P-value
**Species**					
Horse	396	61	15.40	1.00	
Mule	132	5	3.79	0.10 (0.00, 2.38)	0.156
**Educational status of owners**					
Illiterate	120	14	11.67	1.00	
Primary school	292	39	13.36	2.52 (0.37, 17.31)	0.349
High school/above	116	13	11.21	4.79 (0.61, 37.61)	0.136
**Age of animal**					
≤6Yrs	141	23	16.31	1.00	
>6Yrs	387	43	11.11	0.77 (0.18, 3.32)	0.724
**Body condition**					
Poor	190	25	13.16	1.00	
Medium	250	33	13.20	0.29 (0.06, 1.30)	0.106
Good	88	8	9.09	0.88 (0.14, 5.42)	0.889
**Housing**					
Separate	469	22	4.69	1.00	
Shared	59	44	74.58	34.16 (2.47, 473.36)	0.008
**Preexisting wound**					
No	394	17	4.31	1.00	
Yes	134	49	36.57	7.88 (2.08, 29.77)	0.002
**Sharing of Harness**					
No	461	17	3.69	1.00	
Yes	67	49	73.13	226.44 (14.04,3651.77)	0.000
**Feeding/Watering**					
Separate	487	39	8.01	1.00	
Shared	41	27	65.85	0.09 (0.01, 1.33)	0.080
**Grooming practice**					
No	176	9	5.11	1.00	
Yes	352	57	16.19	37.09 (2.38, 578.16)	0.010
**Mingling with other cart animals**					
No	423	5	1.18	1.00	
Yes	105	61	58.10	175.18 (22.95,1337.27)	0.000

In the final risk factor model, a statistically association was observed between the prevalence of the EZL and potential risk factors such as housing condition, harness practices, mingling with other cart animals and preexisting wound presence (P < 0.05) (Table 4). Animals that share a common house, with pre-existing wound, mingle with other cart animals and share harnessing were more at risk of being exposed to the disease than animals that were kept in a separate housing, with no pre-existing, mingle with other cart animals, and animals that do not share harness, respectively.

**Table 4:** The final fitted model for risk factors of epizootic lymphangitis.

Risk factor	Total sampled	Number of Positive	Prevalence (%)	POR (95% CI)	P-value
**Housing**					
Separate	469	22	4.69	1.00	
Shared	59	44	74.58	12.23 (1.86, 80.57)	0.009
**Preexisting wound**					
No	394	17	4.31	1.00	
Yes	134	49	36.57	9.49 (2.59, 34.82)	0.001
**Sharing of Harness**					
No	461	17	3.69	1.00	
Yes	67	49	73.13	109.42 (8.95, 1337.21)	0.000
**Mingling with other cart animals**					
No	423	5	1.18	1.00	
Yes	105	61	58.10	54.51 (9.72, 305.87)	0.000

## 4 Discussion

In this study the disease EZL has been detected in cart horses and cart mules in 2 of 4 towns sampled in Central and South Gondar zones. Two districts where the disease was not detected (Amba Giorgis and Debre Tabor town) were cold highland towns (with average altitude of above 2700 m.a.s.l), which shows that the disease is rare in cold area as has been also demonstrated by previous studies elsewhere [11]. Ameni [11] noted that the disease is endemic in hot, humid areas with an altitude ranging from 1500 to 2300 m.a.s.l and few or no cases were detected in dry-windy and very cold areas of the country. Hot and humid climates promote the survival of the environmental form of the causative agent and also favor the breeding of flies, which could play a role in the mechanical transmission of EZL [13, 15].

The prevalence of the EZL in affected towns was found high with overall prevalence of 12.50%. It was higher in Gondar (19.93%) where only horses were used for cart pulling than in Woreta town (5.75%) where only mules were used for cart business. These variations might be attributed due to the difference in species in which horses might be more susceptible than mules [14]. In addition, the cart equine population in Gondar is high which might contributed for the wide spread and maintenance of the disease in the cart animal population [11, 13].

The prevalence in cart horses determined in this study (19.93%) was in close agreement with the prevalence reported in other parts of Ethiopia such as 21% in Nazerath, 18% in Shashemene, 20% in Robe [11]. Prevalence's that are higher than determined in this study were reported in Mojo (39.1%) by Ameni and Siyoum [20], Bati (32.5 %) and Debre-Zeit (30%) by Ameni [11]. This could be due to the differences in the climatic condition, season of the study and level of attention given for controlling and prevention of the disease.

Majority of the EZL disease studies conducted in Ethiopia focused on cart horses, only two EZL prevalence studies were conducted in cart mules in the country; Ameni and Terefe [36] in western Ethiopia and Meselu *et al.* (22) in Bahir Dar town. The prevalence information documented for mule in this study therefor help to improve the information gap about the situation of the disease in cart mules in the country. The prevalence in cart mules in Woreta town determined in this study (5.75%) was lower than the prevalence of 21% in Bako and Ejaji towns in western Ethiopia reported by Ameni and Terefe [36]and the 32.84% prevalence in Bahir Dar town reported by Meselu *et al.* [22]. This could be due to the differences in the mule population, which was higher in the latter towns.

The characteristic EZL lesions were detected most frequently on limbs, then chest wall, belly area, face and neck region, perineum, inguinal and back region in order of frequency of occurrence. This was in line with the previous reports of Ameni and Terefe [36], Ameni [37] and Meselu *et al.* [22], in which the lesions were mostly confined in areas prone to trauma caused by inappropriate harnessing activities.

Analysis of potential risk factors for the disease revealed that factors such as housing, pre-existing wound, sharing of harness and mingling with other cart animals were significantly associated with the disease prevalence. However, the confidence interval for odds ratios were too wide (Table 4). Among the main reasons for wide confidence intervals are small sample size, complete separation and collinearity [38], which we couldn’t observe in this study. We think these wide confidence intervals could be due to relatively small number of animals in one of the categories of the categorical risk factor variables.

Animals with pre-existing wound were more likely to be affected by EZL compared to cart animals with no previous wound. This is because the causative agent gains direct entry point into the skin and also the wound attracts flies to that area that may act as a mechanical spreader of the agent [12].

The risk of EZL positivity was found much higher in animals that were kept in communal housing than those kept under separate housing. This finding was in line with the report of Ameni [11], who found significantly higher prevalence in animals managed under common shade. Communal shades facilitates the direct contact of the traumatized skin of the naive susceptible animals with agent sources such as infected pus, nasal or ocular excretion of the diseased animal, and facilitates infection spread [14, 39].

The study also demonstrated that sharing of harness increased risk of EZL. This is in agreement with previous studies such as Meselu *et al.* [22], and Mesafint *et al.*, [21]. Sharing of harness, grooming kit and other utensils increase the chance of contact of the causative agent from the diseased animal to the naive animal, since *Histoplasma capsulatum* var. *farciminosum* is highly resistant to the effects of physical and chemical agents, which can survive for longer time in inanimate objects [15].

Higher prevalence was seen in animals mingle with other cart animals in cart stations than in animals that don't mix in the cart stations. Similarly, Ameni [11] reported a higher prevalence in cart horses gathered in cart stations. This is because EZL is highly contagious, spreads most readily where large numbers of animals are come together [13, 15].

There are some limitations in this study that need mentioning. The prevalence was determined on based samples from clinically affected animals and as such the prevalence refers to only the clinical prevalence of the disease. But use of advanced laboratory diagnostic tests such as polymerase chain reaction indicated the existence of the subclinical form of the disease [40].

Most of the management risk factors explored in this study were analyzed as binary variable (‘yes’ or ‘no” or ‘present’ or ‘absent’). But these management practices might occur in continuum from non-extent to very regular and collapsing these into two categories as 'present' or 'absent' might introduce subjectivity. The risk factor variable pre-existing wound was determined based on owners' information which might introduced bias. In light of these limitations a follow up study which addresses the stated limitations would help to validate the risk factors association found in this study.

## 5 Conclusion and recommendations

This study demonstrated that EZL is a prevalent disease in cart horses and mules in the study towns which have mid altitude (ranging 1800–2300 m.a.s.l.) and warmer climate (average annual temperature of about 20 °C). The study identified communal housing, pre-existing traumatic wound, sharing of harness and mingling of cart animal as important risk factor for the disease. The high prevalence of this disease in the affected towns endangers the livelihood of the cart animal owners and welfare of the cart animals, and warrants initiation of a control strategy. The identified risk factors were related to cart animal management, most of which are not onerous to improve. Hence improving the management of cart pulling animals focusing on the identified risk factors is suggested to ameliorate the EZL problem in the study area.

## Declarations

### Author contribution statement

Amsalu Misgie Molla: Performed the experiment; Analyzed and interpreted the data; Wrote the paper.

Wudu Temesgen Jemberu: Conceived and designed the experiment; Analyzed and interpreted the data; Wrote the paper.

Tewodros Fentahun: Conceived and designed the experiments; Contributed reagents, materials, analysis tools or data.

### Funding statement

The study was funded by University of Gondar with grant letter [VRCS 15/2017].

### Data availability statement

Data included in article/supp. material/referenced in article.

### Declaration of interests statement

The authors declare no conflict of interest.

### Additional information

No additional information is available for this paper.

## Appendix A Supplementary data

ANNEXES ANNEXES
