# Supplementary material for: Prevalence and risk factors of epizootic lymphangitis in cart pulling horses and mules in Central and South Gondar zones, Amhara region, Ethiopia
Source: Heliyon. 2022 Jul 19;8(8):e09939. doi: 10.1016/j.heliyon.2022.e09939 (PMC9343934; doi:10.1016/j.heliyon.2022.e09939)
Supplement: ANNEXES [file mmc1.docx]

# ANNEXES

Annex 1: Classification of epizootic lymphangitis cases in to three severity stages (skin form)

| **Severity stage** | **Description** |
| --- | --- |
| Mild | Cases with a cutaneous nodule and lesions are evident in only one body area, detectable either visually or via palpation. |
| Moderate | Case with clinical lesions characterized by the formation of line of infection along the lymphatic consisting of 2-3 nodules and /or ulcers oozing serous fluid. Lesions are distributed all over body and moderate cording is seen on the limb |
| Severe | Cases with extensive abscessation and ulceration of the superficial lymphatic vessels, coalescence of adjacent ulcers and multiple coalescent nodules appear over all four limbs and extensive lesions appear on the face, nodules and “cording” of the lymphatic vessels with involvement of any two or more parts of body. |

Source: Adapted from Endebu [33]

Annex 2: Gram staining procedure [17]

(1) Make a very thin smear of the material on a clean, grease-free glass slide.

(2) Dry in air.

(3) Fix the smear by flaming the slide.

(4) Add gentian violet to cover the smear and leave undisturbed for 1 minute.

(5) Drain off the gentian violet by tilting the slide and rinse in flowing tap water, taking care that the water flow does not fall directly on the smear.

(6) Add Gram’s iodine solution to cover the smear and leave for 1 minute.

(7) Rinse with water in the same way as above.

(8) Decolorize with acetone for about 1-3 seconds.

(9) Quickly rinse again with water.

(10) Counter stain with safranin for 30 seconds, and rinse in water.

(11) Dry in air, and observe under oil immersion (100 x).

**Observation:** Observe the Gram’s reaction (positive or negative), size, shape and arrangement of elements.

Annex 3: Data collecting format during clinical examination and risk factor assessment

**Owners’ name (Optional)** ___________________________________________

Address: Zone_____________________Woreda/Town____________Kebele_______

Village ______________ Tel_________________________ Date______________

1. **Clinical Examination of the Animal**
   1. Body condition score in 1-9 scale ________________________________.
   2. Clinical status (Positive or Negative) _______________________________.
      1. If positive(+ve);
   - Date of beginning of the disease __________________________________________.
   - Lesion severity (Mild, Moderate, Severe) ­___________________________________.
   - Location of lesion ______________________________________________________.
   - The lesion is: A) progressing B) regressing
   - Type samples taken ______________ sample No ____________________
   - Sample result __________
2. **Risk Factors Assessment**
   1. **Owner Profile**

- Educational status of the owner as illiterate, read and write (primary), or high school and above )______________________________.
  1. **Animals (Horse or Mule) Biodata**
- Species_____________ Age_________.
- Year of service in cart work______, when and from where it is acquired in the current owner? ______________________________________________________________.
- Presence of pre-existing wounds and trauma; A)Yes B) no
  1. **Management activity data**
- Number of horses/mules owned together? ____________________.
- Whether other horses/mules are infected (how many are infected by EL)? ________
- Did you have epizootic lymphangitis affected horses/mule before this study? A) Yes B) No; if yes, did you keep the affected and the healthy cart horses separately? i)Yes ii) No
- Does your closest cart horse owner have EL infected horse/mule? i) Yes ii) No, if yes did the animals meet in village? A) Yes B) No
- Did you share harnessing materials with other infected horses/mules? A) Yes B) No
- Did you share harness with other non-infected horses? A) Yes B) No
- Did you share whips with other infected horses? A) Yes B) No
- Did you share whips with other non-infected horses? A) Yes B) No
- Did you share washing brush/other cleaning material to both the non-infected and infected horses of your own or with somebody else? A) Yes B) No
- What is the Washing/cleaning (grooming) frequency of your animal? ______________/week/month.
- Feeding, watering and housing activities? A) separated B) together
- Does your cart horse meet or assembled together with EL affected horse in cart station, in road or market? A) Yes B) No
  1. **Environment Risk Factor**
- Altitude of the town district in m. a. s. l.? _______________.
- Average annual temperature (^0^C)? ____________.
- Average humidity? _________.

Annex 4:

Body condition scoring of equines

| **Score** | **Description** |
| --- | --- |
| **1** | **Poor:**  The animal is extremely emaciated. The backbone, ribs, hipbone and tail head projecting prominently. Bone structure of the withers, shoulders and neck easily noticeable. No fatty tissue can be felt. |
| **2** | **Very Thin:**  The animal is emaciated. Slight fat covering over vertebrae. Backbone, ribs, tail head and hipbone are prominent. Withers, shoulders and neck structures faintly discernible. |
| **3** | **Thin:**  Fat build up about halfway on vertebrae. Slight fat layer can be felt over ribs, but ribs easily discernable. Tail head is evident, but individual vertebrae cannot be visually identified. Hipbones cannot be seen, but withers, shoulders and neck are emphasized. |
| **4** | **Moderately Thin:**  Negative crease along back. Faint outline of ribs can be seen. Fat can be felt along tail head. Hipbones cannot be seen, but withers, shoulders and neck not obviously thin. |
| **5** | **Moderate:**  Back is level. Ribs can be felt but not easily seen. Fat around tail head beginning to feel spongy. Withers appear rounded. Shoulders and neck blend smoothly into body. |
| **6** | **Moderately Fleshy:**  May have slight crease down the back. Fat over ribs feels spongy. Fat around tail head feels soft. Fat beginning to be deposited along the side of the withers, behind the shoulders and along the sides of the neck. |
| **7** | **Fleshy:**  A crease is seen down the back. Individual ribs can be felt, but noticeable filling between ribs with fat. Fat around tail head is soft. Noticeable fat deposited along withers, behind shoulders and along neck. |
| **8** | **Fat:**  Crease down back is prominent. Difficult to feel ribs due to fat in between. Fat around tail head very soft. Area along withers filled with fat. Area behind shoulder filled in flush with the barrel of the body. Noticeable thickening of neck. Fat deposited along inner thigh. |
| **9** | **Extremely Fat:**  Obvious crease down back. Patchy fat appearing over ribs. Bulging fat around tail head, along withers, behind shoulders and along neck. Fat along inner thighs may rub together. Flank filled in flush with the barrel of the body. |

Source: Henneke *et al.* [35]

**Annex 5**


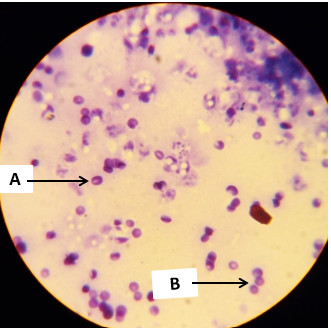


Annex figure 1. An example of yeast positive smear; arrow heads indicting A) individual yeast cell and B) yeast cells in groups under 100X (oil immersion) magnification.
